# Knowledge about stem cell sources and obstacles in donation of bone marrow and peripheral blood stem cells: a cross-sectional survey from Ha’il city to track the prospects of regenerative medicine in Saudi Arabia

**DOI:** 10.1186/s40780-023-00299-6

**Published:** 2023-08-14

**Authors:** Asma Ayyed AL-Shammary, Sehar un-Nisa Hassan

**Affiliations:** Department of Public Health, College of Public Health and Health Informatics, University of Ha’il, Ha’il, 81451 Kingdom of Saudi Arabia

**Keywords:** Regenerative medicine, Stem cell donation, Willingness, Registration, Awareness, Knowledge

## Abstract

**Background:**

Promoting stem cell donation behaviors could be crucial in advancing stem cell-based treatment, research and improving public health in Saudi Arabia. Donation of stem cells can be considered an act of social welfare just like blood donation because stem-cell-based therapies are emerging as a hope for those suffering from chronic health conditions and/or terminal illnesses.

**Aim:**

This study aims at assessing levels of awareness about sources of stem-cells, donor organizations and predictors of stem cell donation behavior in target population.

**Methods:**

The study employed a cross-sectional online survey method. The study sample comprises 1325 educated Saudi people living in Ha’il city. The survey questionnaire collected data about respondents’ demographic background, awareness about various sources of stem cells and stem-cell donor registries, willingness to donate stem cells, registration status and obstacles in stem cell donation registration. Percentages, Chi-square analysis and Odd Ratios were computed to analyze the data.

**Results:**

In this sample, (*n* = 696; 52%) were males and (n = 629; 48%) were females. Although (*n* = 1308; 98%) percent of respondents reported willingness to donate stem cell, less than one percent (*n* = 6; 0.5) were registered with Saudi Stem Cell Registry. Over 50% of respondents hold inaccurate perceptions about sources of stem cell. Odd Ratio (OR) values from binary logistic regression model identified four factors as significant predictors of non-registration status. These included (i) unaware about donor agencies and procedures (OR = 10.07; *p* < 0.05), (ii) unaware about possibility to donate stem cells (OR = 8.08; *p* < 0.05) (iii) concerns about impact on health (OR = 10.01; *p* < 0.05) and (iv) have health issues that does not permit donation (OR = 10.50; *p* < 0.05).

**Conclusion:**

Stem cell registrations can be enhanced through appropriate health education programs that focus on increasing awareness about donation procedures, trustworthy donor organizations and reducing people apprehensions related to donation.

## Introduction

The basic facts about the sources of stem cells, authorized donor agencies and stem cell donation procedures need to be widely known and understood by the various segments of the population to prevent health risks and promote donation behaviors. Regenerative therapies have demonstrated significant progress in alleviating terminal illnesses and promoting better health for patients with chronic diseases. Nonetheless, there are emerging challenges such as access to safe and medically approved treatments, social justice and cost implications. Both popularity and speculations about stem-cell-based therapies are intensifying in Saudi Arabia and other Middle East countries. Previous literature has identified that infodemic around stem cell therapies is appearing as a key challenge that may increase the vulnerability of patients to the harms of unethical approach and malpractices in stem-cell based treatments [1]. Additionally, healthcare providers face significant challenges in finding matched donors for stem cell transplant in Saudi Arabia and it is recommended to have local registry to improve the prospects of finding a matched donor [2]. Although Saudi Arabia claims to be the first country in the Arab world to have more than 10,000 stem cell donors, it is currently underperforming in this domain as compared to European and other developed countries. According to available statistics, the rates of registered stem cell donors are just around one per cent in Saudi Arabia [3]. The underlying factors responsible for this scenario could be lack of awareness about basic facts such as sources of adult stem cells, procedures of adult stem cell retrieval, donation risks and outcomes and information about authorized local and international donor agencies etc.

One study from the capital city of Saudi Arabia (Riyadh) demonstrated that 58% of the respondents never heard about stem cell donation and 33% were not willing to register for stem cell donation [4]. Those who expressed willingness to register had insufficient knowledge about stem cells. This study did not collect any follow-up data to demonstrate the rates of registration for donation among people who expressed willingness to donate stem cells during the campaign. Another study from the same city demonstrated that 19% of respondents from the general population had no idea about stem cells [5]. One study from the Mecca region of Saudi Arabia assessed the acceptance and refusal rates for stem-cell registration in a sample of around 500 participants. Findings showed that only 1.8% of respondents were registered with stem cell donation organization despite 96% were willing to donate stem cells in case if it is needed for the treatment of a family member [3]. This shows that their willingness to donate stem cells is largely conditional. Moreover, such donation behaviors will not serve the purpose because the growing body of scientific literature suggests the need for ethnically diverse stem cell donors to accomplish the beneficial outcomes of stem-cell based treatment [6].

Promoting stem cell donation behaviors could be crucial in advancing stem cell-based treatment, research and improving public health in Saudi Arabia [7]. Donation of stem cells can be considered an act of social welfare just like blood donation because stem-cell-based therapies are emerging as a hope for those suffering from chronic health conditions and/or terminal illnesses. Donating stem cells is, therefore, emphasized by public health experts, donor agencies and stem-cell research organizations to gain beneficial outcomes from this exciting young science. Series of surveys conducted in Switzerland demonstrated that both perceptions and knowledge-related factors were the key barriers to registration on the Swiss Blood Stem Cell Registry [8]. In Saudi Arabia, patients primarily rely on international donor centers to recruit stem cells for treatment purposes [9]. The inconsistencies in terms of compliance with ethical and medical regulations for stem cell retrieval procedures and transplants [10, 11], may pose health risk for the patients and cost implications for the families who are not aware about basic facts related to regenerative medicine.

The review of local studies demonstrated, to date, there is no population based large-scale survey available from Ha’il region of Saudi Arabia that could educate and inform stakeholders on this issue. The existing empirical evidence is insufficient to design health education policy and health awareness programs that are needed to promote stem cell donation behaviors among people in this region. This is because available studies recruited small sample sizes from general population and restricted to the cities of Riyadh and AlHassa only [5, 12, 13]. Other local studies assessed the knowledge and attitudes of medical professionals and nursing students [14, 15]. In our prior national study, we found that rates of registration for donation umbilical cord stem cells among pregnant women in Saudi Arabia was just around one percent [16]. It is, therefore, of prime significance to study current levels of knowledge about sources of stem cells and donor agencies among different sub-populations from geographical regions which have not been explored earlier. Identification of factors associated with willingness to donate stem cells and any obstacles faced by them in registering with Saudi Stem Cell Donor Registry will be useful to track the future pathways of regenerative medicine in Saudi Arbia.

Regenerative medicine is an emerging and a very specialized area of public health knowledge in Saudi Arabia; it was therefore a realistic and practical approach to target educated people in this first survey of this series. to have the capacity to influence the perceptions of the community thus current research focused on this segment of Saudi population living in Ha’il region.

This study aims at determining levels of awareness, willingness to donate bone marrow and/or peripheral blood stem cells, registration status and factors associated with non-registration in the target population of this study. Following are some specific research questions answered by employing a cross-sectional survey design conducted in Ha’il city of Saudi Arabia.

- Are people accurately aware about sources of stem cells in target population?
- Are people in target population aware of Saudi Stem Cell Registry and International donor organizations?
- What is the most common means of information about sources of stem cells and donor agencies?
- What are the rates of willing to donate stem cells and registration with Saudi Stem Cell Registry for stem cell donation in target population?
- What type of hinderances significantly impact the intention to donate and registration status?

The study will add to the existing literature by collecting data from the geographical region which has not been investigated in previous local studies. Lack of awareness regarding basic facts related to sources of stem cells, stem cell donor agencies and procedures could increase the risk of being wronged by non-credible organizations. The insight gained from the findings will be useful in the formulation of health education policy and action plans to raise awareness, address barriers and promote stem cell donation registration at local, national and regional level.

## Methods

### Geographical region of study

The current study was conducted in Ha’il city of Saudi Arabia. Haʼil city is in the northwest of Saudi Arabia and the city covers an area of about 630 sq/km. The current population is around three hundred thousand with more than 80% Saudi nationals [17].

### Target population

The target population for this cross-sectional study were male and female adult Saudi nationals who have completed at least 12 years of formal education, with adequate physical and mental capacity to respond to a self-report online questionnaire. There were theoretical, technical and practical reasons for choosing this sub-population of Ha’il city as a target population in current study. Educated people are the gatekeepers of the community and targeting them for public health interventions and health education program provides a technical advantage to mobilize communities at large. This study is conducted as part of the series of studies and collected data through self-report electronic questionnaire. The survey was restricted to those who have apparent capacity to read, understand and respond to this electronic survey to retain the validity of data.

The specific details related to socio-demographic profile of target population are: Saudi nationals; male or female with 18 years and above age, residents of Ha’il city, completed 12 years of formal education, active users of internet/social media tools with sufficient proficiency to complete a self-report electronic questionnaire in Arabic or English version.

### Study sample

The sample size for the current study was estimated according to the statistical formula for cross-sectional survey research [18]. The estimated sample size was found to be 945 respondents by choosing the level of significance (α) at 5% and desired levels of precision (d2) at 3% with the z-score corresponding value of 1.96. We collected around 3% of additional data to compensate for the loss of data due to invalid forms or missing responses. A total of (*N* = 1410) individuals accessed the survey link for the questionnaire, out of which (*n* = 11;0.7%) did not consent to participate in the study, (*n* = 32;2.2%) did not live in Ha’il city and (*n* = 42;3%) were not Saudi nationals. The response rate (RR) for the study was (93%) and the final sample comprised of (*n* = 1325) individuals.

### Survey instrument

The data was collected on an electronic version of a survey questionnaire presented as different sub-sections.

- (i) Screening Items: The first part of the electronic survey presents information about study objectives and comprises of four screening items to recruit participants according to the inclusion/exclusion criteria. Those who met the inclusion/exclusion criterion proceeded to main study questionnaire.
- (ii) Main Study Questionnaire: The main survey questionnaire has the following sub-sections.
- (a) The demographic variables included gender, age, years of education and field of education.
- (b) Participants were asked six distinct questions to identify whether (i) blood, (ii) fatty tissue, (iii) spinal cord, (iv) umbilical cord, (v) bone marrow, and (v) brain are sources of stem cells for donation. The items were followed by ‘Yes/No/Do not Know’ response categories.
- (c) Participants were asked to report primary means of information about stem cells with response categories of (i) formal education, (ii) social media/internet and (iii) others with a blank space to mention the source.
- (d) Two direct close-ended questions assessed respondents’ awareness about donor agencies. The first question was “Do you know about the Saudi Stem Cell Donor Registry or other donor agencies in Saudi Arabia to collect stem cells from donors?” and “Are you aware of international donor agencies which collect stem cells from donors? The response categories were ‘Yes/No’ for both items.
- (e) The respondents were referred to the website of Saudi Stem Cell Donor Registry in King Faisal Specialist Hospital and Research Centre [19] to share basic information and understand the commitment before becoming a stem-cell donor. This registry primarily retrieves stem cells through bone marrow or peripheral blood. This information is followed by two direct questions *“Are you willing to donate stem cells?*” and *“Are you currently registered with the Saudi Stem Cell Donor Registry*? The response categories were ‘Yes/No’ for both items.
- (f) The last part of the survey questionnaire inquired about the six underlying factors that may associate with non-registration with donor registry. These are (i) not aware about possibility of stem cell donation (ii) don’t believe in stem cell donation (iii) health issues restrict them to donate (iv) concerns about the side impacts of stem cell donation on their health, (v) unaware about donor agencies/procedures and last one is (vi) believe that religion does not permit for stem cell donation. Each underlying factor was assessed by a distinct statement and followed by a True/False option.

#### Process of the adaptation and validation of survey instrument

The survey instrument was adapted by following the guidelines in the development and adaptation of online study questionnaires for quantitative research [20]. One of the team members on this research project has professional knowledge and experience in the field of psychometrics and epidemiological research and other team member hold expertise in field of stem cell research. The items on the main study questionnaire were adapted from the study instruments used in prior studies [4, 8, 21]. These survey items were critically reviewed by an expert in epidemiological research and regenerative medicine research. To determine the validity and consistency of items on survey questionnaire, each item was assessed on indicators of clarity, conciseness, biasness, and double-barreled wording by two independent field experts. During this process the items on which 80% consensus of field experts was obtained were retained in the final survey. This is to ensure the content and construct validity of the survey. The survey items were initially developed in English language and were translated by two colleagues in the public health department who are well-versed in both languages. They independently completed the translation work through translation-back-translation process. Pilot testing was completed on both English and Arabic online versions of the questionnaire in target population and minor suggestions related to formatting were incorporated.

### Data collection procedure

A data collection procedure strategy was devised to ensure that electronic survey links generated to collect data for this survey are accessed by the target population. The survey links were distributed with help of volunteers during the variety of local events and activities that were conducted in Ha’il city between 8 and 20th of January 2023. They include but are not limited to medical camps, community health seminars, local sports festival, cultural festivals, on campus professional conferences and social gatherings etc. Generally, in Saudi Arabia and more specifically in Ha’il city, these events and community activities are well-planned and conducted under the jurisdiction of the Ministry of Health, Ministry of Education and Ministry of Culture. These events and activities provided an optimal way to access the target population as these activities include both health education and entertainment component and are well-marketed by the organizers. People from different segments of the general population and disciplines are involved in these activities either as participants or visitors.

During these events the electronic survey link was distributed through various digitals and social media tools. 99% of the population in Saudi Arabia are Internet users, therefore it was appropriate to employ digital tools for distribution of survey link and it served multiple purposes (large sample size in a short period of time, privacy and direct transformation of data to statistical software’s for analysis). Participation in the study was voluntary and all respondents before proceeding to the main study questionnaire signed an electronic informed consent form with key information about study aim and objectives.

### Ethical approval

The study protocol was reviewed by the Ethical Review Board at University of Ha’il and approved (H-2022354) dated 31/10/2022. All participants were provided information about study before obtaining consent, the confidentiality and anonymity of data was maintained during all stages of study.

### Statistical analysis

The electronic survey link provides the raw data on Excel sheets. The raw data is coded and transformed to IBM SPSS for statistical analysis. Descriptive analysis (frequency and percentage values) and inferential analysis (chi-square analysis and multiple logistic regression analysis) were conducted to answer research questions for this study. Pie-chart and Bar graphs display descriptive findings. Chi-square test determines significance of bivariate association between background variables and willingness to donate/registration for donation. Odd Ratios (OR) are computed to determine the influence of various factors in non-registration for stem-cell donation among those willing and not willing to donate. For the interpretation of findings on inferential analysis, the chosen level of p-value significance at *p* < 0.05.

## Results

The study sample comprises of 1325 respondents and all of them were Muslims, and Saudi nationals. Other demographic characteristics of the study sample are shown in Table 1.

**Table 1:** Descriptive data on demographic study variables (*n* = 1325)

Variables	Categories	Frequency (n)	Percentage (%)
**Gender**	Female	629	48%
	Male	696	52%
**Age**	18–29 years	212	16%
	30–39 years	517	39%
	40–49 years	400	30%
	50 yrs & above	196	15%
**Field of Education**	Social Sciences and Humanities	425	32%
	Medical Science /Nursing/ Pharmaceutical Sciences	318	24%
	Engineering/Computer Science	310	23%
	Others (Health Management/ Public Health/Biological Sciences/ Environmental Sciences/ Natural Sciences)	272	20%

**Fig. 1:**
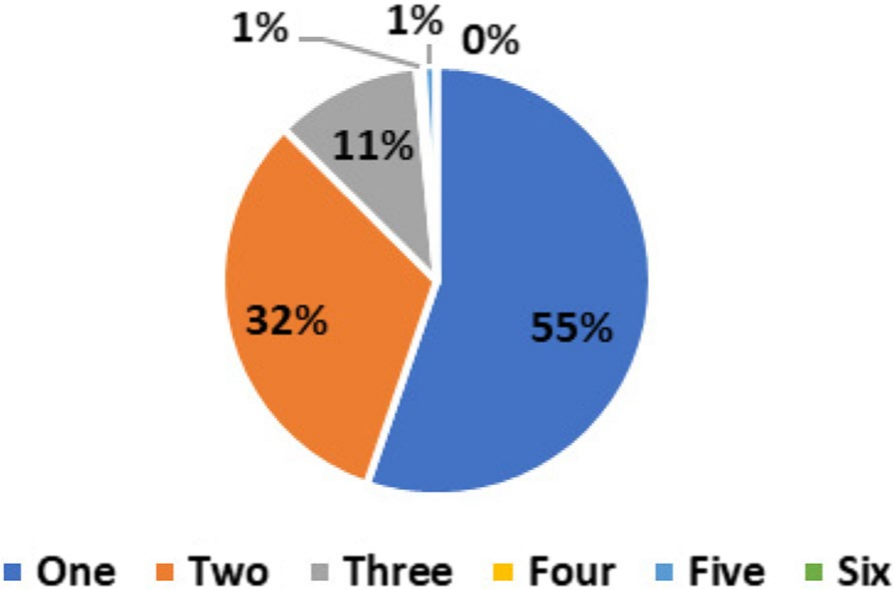
Participants were asked six distinct questions to identify whether (i) blood (ii) fatty tissue (iii) spinal cord, (iv) umbilical cord (v) bone marrow, and (vi) brain are sources of stem cells for donation. Figure 1 reports the percentage of correct responses given by respondents to these six questions (*n* = 1325)

Just around (*n* = 13; 1.0%) of the respondents correctly perceived that bone marrow is a source of stem cells. Although spinal cord is not a source of stem cells but (*n* = 1310; 99%) of respondents considered it as a source of adult stem cells. Besides fatty/adipose tissue despite being an important source of stem cells, only (*n* = 575; 43%) of respondents. Also, only (*n* = 11; 1%) endorsed that blood is a source of stem cells and (*n* = 152; 11%) of participants considered the brain a source of stem cells. Most respondents (*n* = 1313: 99%) have correctly reported that umbilical cord is a source of adult stem cells (Table 2). The frequency and percentage of respondents who gave number of correct answers on these six questions which inquired about sources of stem cells is shown in Fig. 1. The results show that (*n* = 734; 55%) of participants gave only one correct answer out of six question that asked about different sources of stem cells. (*n* = 435; 32%) gave four correct answers followed by (*n* = 147; 11%) who gave three correct answers. Only (*n* = 9; 1%) of participants gave correct answers on five items and four items, respectively.

**Table 2:** Descriptive data on participants perceptions about sources of stem cells, awareness about donor agencies and means of information (*n* = 1325)

Variables	Categories	Frequency (n)	Percentage (%)
**Sources of stems cells (Yes)**	Bone Marrow	13	1.0%
	Blood	18	1.0%
	Brain	152	11%
	Fatty Tissue	575	43%
	Spinal Cord	1310	99%
	Umbilical Cord	1313	99%
**Aware about local registries for stem cell donation**	Yes	877	66%
	No	448	34%
**Aware about international registries for stem cell donation**	Yes	141	11%
	No	1184	89%
**Direct interaction with any stem cell donor organization**	Yes	139	10%
	No	1186	90%
**Means of information about stem cells**	Formal Education	563	42%
	Social Media/Internet	762	58%

Table 2 also shows that percentage of those who were aware of local stem cell donor agencies were (*n* = 877; 66%) and those aware of international donor registries for stem cell donation were (*n* = 141; 11%). Among those who were aware of either national or international donor agencies (*n* = 139; 10%) reported direct interaction with any donor organization. Most of the respondents (*n* = 762; 58%) reported that social media/online sources are the major sources of information on stem cells (Table 2).

**Fig. 2:**
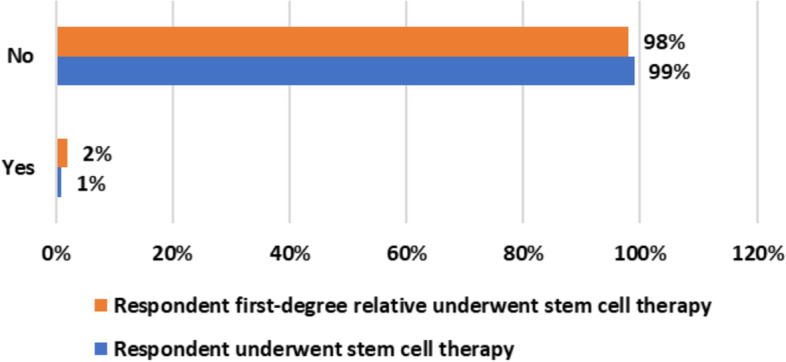
Respondents were referred to website of Saudi Stem Cell Registry [19] which provides basic information about stem cell donation and stem cell therapy both in English and Arabic languages. Figure 2 shows the percentage of participants who themselves or their first-degree relative underwent stem-cell based therapy (*n* = 1325)

Figure 2 shows that only (*n* = ;1%) of respondents in this study sample ever underwent stem cell therapy and (*n* = ;2%) reported their first-degree relatives underwent stem cell therapy.

**Fig. 3:**
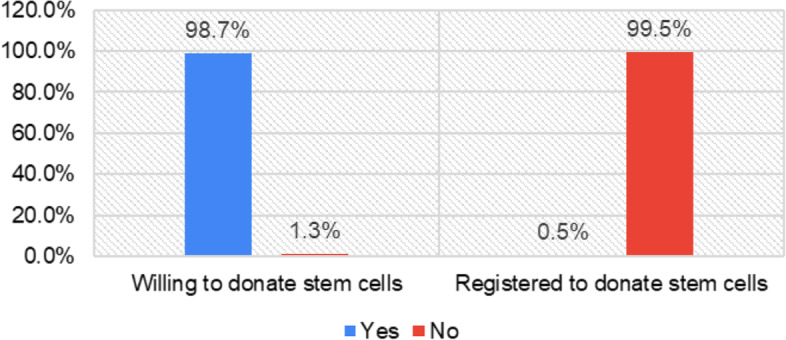
In Saudi Arabia, the donor registries are for Bone Marrow Stem Cells/Peripheral Blood Stem. The respondents were referred to the website of Saudi Stem Cell Registry [19] which provides complete information related to stem cell donation and donor commitment. Figure 3 shows the percentage of participants who reported willingness to donate bone marrow/peripheral blood stem cells and percentage of participants who are currently registered with Saudi Stem Cell Registry for donation of stem cell (*n* = 1325)

Figure 3 shows that (*n* = 1308; 98.7%) of participants showed their willingness to donate stem cells, however, only (*n* = 6; 0.5%) of respondents were registered with Saudi Stem Cell Registry.

Table 3 shows statistically significant association of gender (p < 0.05), age (p < 0.001) and the field of education (*p* < 0.05) with willingness to donate. Those who consider bone marrow and blood are not the sources of stem cells significantly associated with willingness to donate at (p < 0.001). Those who consider spinal cord and umbilical cord are the sources of stem cells significantly associated with willingness to donate stem cells (*p* < 0.001). Awareness about Saudi Stem Cell Registry and direct interaction with any stem cell donor registry was significantly associated with willingness to donate (*p* < 0.01). Direct and indirect exposure to stem cell therapy experience were significantly associated with willingness to donate stem cells (*p* < 0.001). Information retrieved from social media was found to significantly associate with willingness to donate stem cells (*p* < 0.05) (Table 3).

**Table 3:** Chi-square to demonstrate association of study variables with a willingness to donate stem cells and registration status (*n* = 1325)

			Willingness to Donate		Registered for donation	
			Yes	No	Yes	No
Variables	Categories		n (%)	n (%)	n (%)	n (%)
Gender	Female		616 (48%)	13 (76%)	5 (83%)	624 (47%)
	Male		692 (52%)	4 (24%)	1 (17%)	695 (53%)
*Chi-square & p-value significance*			*Χ*^*2*^ = 5.08^*^		*Χ*^*2*^ = 3.10 (ns)	
Age	18–29 years		203 (16%)	9 (53%)	2 (33%)	201 (16%)
	30–39 years		512 (39%)	5 (29%)	2 (33%)	515 (39%)
	40–49 years		397 (30%)	3 (18%)	2 (33%)	398 (30%)
	50 yrs & above		196 (15%)	0 (0.0%)	0 (0.0%)	196 (15%)
*Chi-square & p-value significance*			*Χ*^*2*^ = 18.5^***^		*Χ*^*2*^ = 2.09 (p = ns)	
Field of Education	Medicine/Nursing/Pharmaceutical Sciences		317 (24%)	1 (6%)	0 (0.0%)	318 (24%)
	Engineering/Computer Science/Business Administration		309 (23%)	1 (6%)	1 (17%)	309 (23%)
	Social Sciences and Humanities		418 (32%)	7 (41%)	0 (0.0%)	425 (32%)
	Others (Public Health/Health Management/Environment Sciences/Natural Sciences)		264 (21%)	8 (47%)	5 (83%)	267 (20%)
*Chi-square & p-value significance*			*Χ*^*2*^ = 10.9^*^		*Χ*^*2*^ = 15.07^**^	
Bone Marrow, source of stem cells	Yes		7 (0.5%)	6 (35%)	6 (100.0%)	10 (0.8%)
	No		1301 (99.5%)	11 (65%)	0 (0.0%)	1309 (99.2%)
*Chi-square & p-value significance*			*Χ*^*2*^ = 208.7^***^		*Χ*^*2*^ = 149.07^***^	
Blood, source of stem cells	Yes		10 (1.0%)	8 (47%)	4 (66.7%)	14 (1.1%)
	No		1298 (99%)	9 (53%)	2 (33.3%)	1305 (98.9%)
*Chi-square & p-value significance*			*Χ*^*2*^ = 268.3^***^		*Χ*^*2*^ = 191.84^***^	
Brain, source of stem cells	Yes		148 (11%)	4 (24%)	0 (0.0%)	149 (11.3%)
	No		1160 (89%)	13 (76%)	6 (100.0%)	1170 (88.7%)
*Chi-square & p-value significance*			*Χ*^*2*^ = 2.46 (ns)		*Χ*^*2*^ = 8.81^**^	
Fatty tissues, source of stem cells	Yes		567 (43%)	8 (47%)	6 (100%)	569 (43.1%)
	No		741 (57%)	9 (53%)	0 (0.0%)	750 (56.9%)
*Chi-square & p-value significance*			*Χ*^*2*^ = 0.09 (ns)		*Χ*^*2*^ = 7.86^**^	
Spinal cord, source of stem cells	Yes		1302 (99.5%)	8 (47%)	0 (0.0%)	1307 (99.1%)
	No		6 (0.5%)	9 (53%)	6 (100.0%)	12 (0.1%)
*Chi-square & p-value significance*			*Χ*^*2*^ = 412.9^***^		*Χ*^*2*^ = 128.6^***^	
Umbilical cord, source of stem cells	Yes		1303 (99%)	10 (59%)	6 (100%)	1307 (99.1%)
	No		5 (1.0%)	7 (41%)	0 (0.0%)	12 (0.9%)
*Chi-square & p-value significance*			*Χ*^*2*^ = 311.1^***^		*Χ*^*2*^ = 0.05 (ns)	
Aware about local registries for stem cell donation	Yes		872 (67%) 5 (29%)		4 (67%) 5 (1.0%)	
	No		436 (33%) 12 (71%)		2 (33%) 1314 (99%)	
*Chi-square & p-value significance*			*Χ*^*2*^ = 10.4^**^		*Χ*^*2*^ = 389.02^***^	
Aware about international registries for stem cell donation	Yes		135 (10%)	6 (35%)	6 (100%)	135 (10%)
	No		1173 (90%)	11 (65%)	0 (0.0%)	1184 (90%)
*Chi-square & p-value significance*			*Χ*^*2*^ = 11.06**		*Χ*^*2*^ = 50.61^***^	
Direct interaction with any stem cell donor registry	Yes		133 (10%)	6 (35%)	6 (100%)	133 (10%)
	No		1175 (90%)	11 (65%)	0 (0.0%)	1186 (90%)
*Chi-square & p-value significance*			*Χ*^*2*^ = 11.28^**^		*Χ*^*2*^ = 51.42^***^	
Means of information	Formal Education		560 (43%)	3 (18%)	1 (17%)	562 (43%)
	Social Media/Online Sources		748 (57%)	14 (82%)	5 (83%)	757 (57%)
*Chi-square p-value significance*			*Χ*^*2*^ = 4.35^*^		*Χ*^*2*^ = 1.64 (ns)	
Respondent underwent stem cell therapy	Yes		1 (0.1%)	4 (24%)	4 (68%)	9 (1.0%)
	No		1307 (99.9%)	13 (76%)	2 (32%)	1310 (99%)
*Chi-square p-value significance*			*Χ*^*2*^ = 245.5^***^		*Χ*^*2*^ = 267.6^***^	
Respondent first-degree relative underwent stem cell therapy	Yes		9 (1.0%)	5 (29%)	5 (83%)	9 (1.0%)
	No		1299 (99%)	12 (71%)	1 (17%)	1310 (99%)
*Chi-square p-value significance*			*Χ*^*2*^ = 132.4^***^		*Χ*^*2*^ = 390.2^***^	

^X2=Chi^^−^^square; ^^*p*−value sig^

^***^^p<0.001^

^**^^p<0.01^

^*^^p<0.05, *ns* non−significant^

There were some factors which showed statistically significant bivariate association with registration status (Table 3). Among demographic factors, education was found to be significantly associated with registration status at (*p* < 0.01). Perceptions about bone marrow, blood and spinal cord as sources of stems cell were associated with registration status at (*p* < 0.001). Similarly, perceptions about fatty tissue and brain as sources of stem cells associate with registration status at (*p* < 0.01). Awareness about local and international donor organizations, direct interaction with donor agencies and prior exposure of stem cell therapy were significantly associated with registration for stem cell donation (*p* < 0.001). Having a first-degree relative who underwent stem cell therapy significantly associate with stem cell registration at (*p* < 0.001) (Table 3).

The multiple logistic regression analysis demonstrates that two variables’ perceptions about fatty tissues as sources of stem cells (OR = 1.101; 95% CI = 0.00–1.90; *p* < 0.01) and awareness about Saudi Stem Cell Registry cells (OR = 13.9; 95% CI = 11.04–16.9; *p* < 0.01) appeared as statistically significant predictors for willingness to donate stem cells in a multivariate model. (Table 4).

**Table 4:** Logistic regression to determine demographic, knowledge about sources of stem cells, donor agencies and other predictors of willingness to donate stem cells (*n* = 1325)

		Willingness to Donate		
		B	OR	95% (CI)
Gender (*Ref Female)*	Male	0.11	1.12(ns)	0.14–2.52
Age *(Ref 50 yrs. & above)*	18–29 years	-1.91	0.00(ns)	0.00–1.21
	30–39 years	-1.61	0.00(ns)	0.00–1.61
	40–49 years	-1.71	0.00(ns)	0.00–1.32
Field of Education *(Ref Others……)*	Medicine/Nursing/Pharmaceutical Sciences	1.00	2.72(ns)	0.11–6.17
	Engineering/Computer Science/Business Administration	.776	2.17(ns)	0.09–4.93
	Social Sciences and Humanities	1.59	4.94(ns)	0.01–6.41
Bone Marrow, source of stem cells *(Ref No)*	Yes	.424	1.52(ns)	0.13–1.89
Blood, source of stem cells *(Ref No)*	Yes	-3.47	.031(ns)	.0011.29
Brain, source of stem cells *(Ref No)*	Yes	3.56	3.51(ns)	.341–4.62
Fatty tissues, source of stem cells *(Ref No)*	Yes	7.08	1.101^**^	0.00–1.90
Spinal cord, source of stem cells *(Ref No)*	Yes	.718	2.05(ns)	0.00–3.11
Umbilical cord, source of stem cells *(Ref No)*	Yes	4.07	8.6(ns)	0.01–9.13
Aware about local registries for stem cell donation *(Ref No)*	Yes	8.38	13.9**	11.04–16.9
Aware about international registries for stem cell donation *(Ref No)*	Yes	-.384	.681(ns)	.000–1.23
Direct interaction with any stem cell donor registry *(Ref No)*	Yes	-3.26	.038(ns)	.000–1.39
Means of information *(Ref Social Media/ Online Sources)*	Formal Education	0.63	1.89(ns)	0.13–1.46
Respondent underwent stem cell therapy *(Ref No)*	Yes	-11.08	1.02(ns)	0.00–1.58
Respondent first-degree relative underwent stem cell therapy *(Ref No)*	Yes	3.23	2.55(ns)	0.04–1.31

^**^p < 0.01

^***^p < 0.001; *ns* non-significant, *B* unstandardized regression weight, *OR* Odd Ratio, *95% CI* 95% Confidence Interval

**Fig. 4:**
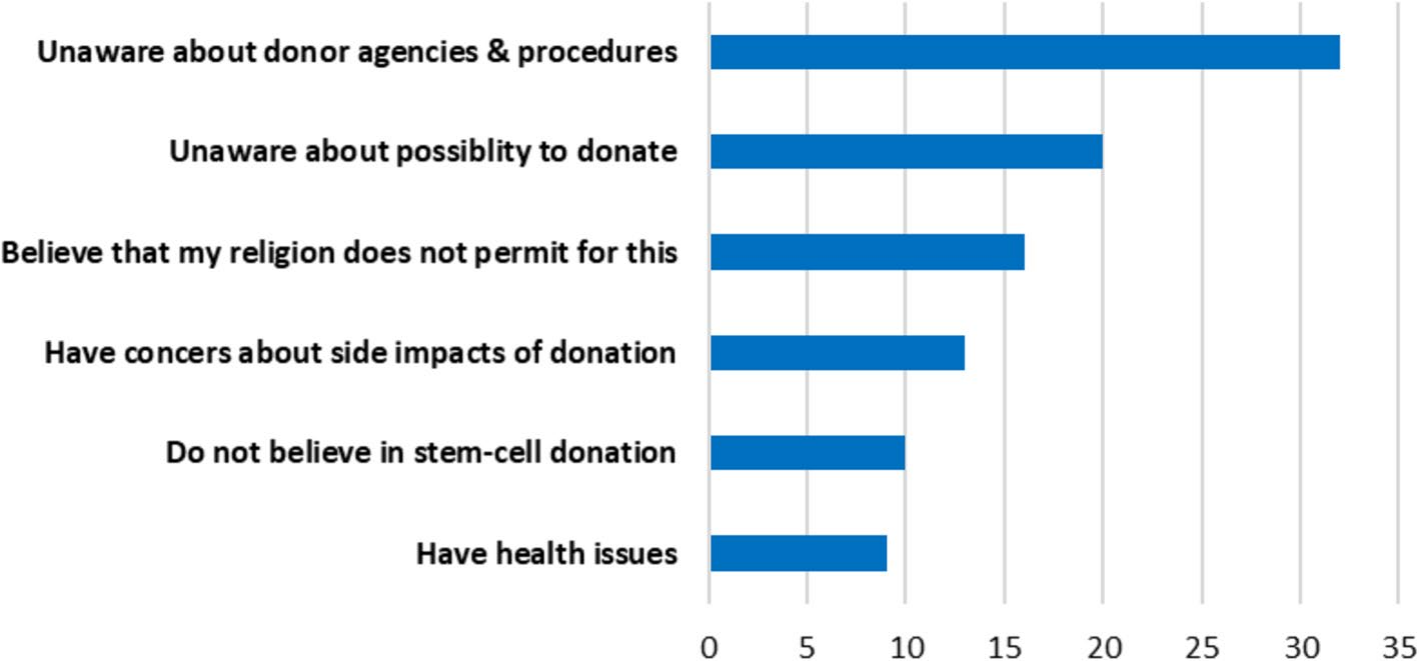
Respondents were inquired about reasons for non-registration with stem cell donor registry. Figure 4 shows the percentage of participants who reported the above-mentioned reasons for non-registration with stem cell donor agencies (*n* = 1319)

A large percentage of respondents (*n* = 1319; 99.5%) were not registered with Saudi Stem Cell Registry or any other organization. Figure 4 shows the factors reported by participants as barriers in registration for stem cell donation.

The percentage values shows that around one-third of respondents reported *‘lack of awareness about donor agencies and procedures”* was the major barrier for non-registration with Saudi Stem Cell Registry or any other organization (Fig. 4). Among other reasons ‘unawareness about possibility to donate’, ‘religious concerns’ and ‘concerns about side impact on health’ were noteworthy reported by 20%, 16% and 13% of respondents respectively.

Table 5 presents the Odd Ratio (OR) values from univariate binary logistic regression analysis to demonstrate the strength of relationship between underlying reasons and non-registration status. Findings show that four factors appeared as significant factors. These included (i) unaware about donor agencies and procedures (OR = 10.7; 95% CI 1.25–12.89; *p* < 0.05), (ii) unaware about possibility to donate stem cells (OR = 8.10; 95% CI = 1.47–9.89) (iii) concerns about impact on health (OR = 10.01; 95% CI 2.98–11.23; *p* < 0.05) and (iii) have health issues that does not permit donation (OR = 10.50; 95% CI 2.05–11.74; *p* < 0.05).

**Table 5:** Univariate binary logistic regression analysis to demonstrate relationship between underlying reasons for non-registration (*n* = 1325)

	**B**	**S.E**	**Wald**	**OR**	**95% CI for OR**
Unaware about donor agencies and procedures	2.37	1.09	4.69	10.7^*^	1.25–12.89
Unaware about possibility to donate stem cells	2.09	0.86	5.80	8.10^*^	1.47–9.89
Thinks that religion does not permit this	2.82	1.55	1.37	2.16	2.01–4.44
Concerns about side impacts of donation on health	2.57	0.87	3.81	10.01^*^	2.98–11.23
Rejection attitudes towards donation	2.18	0.82	7.09	8.91	1.78–9.89
Have health issues and cannot donate for that reason	3.01	0.87	4.88	10.50^*^	2.09-.10.74

^**^p < 0.01

^***^p < 0.001; *ns* non-significant, *B* Unstandardized regression weights, *S.E.* Standard Error, *WaldX*^*2*^ Wald Statistics, *OR* Odd Ratio, *CI* Confidence Interval

## Discussion

The scope of current study is contextualized within the sociopolitical and ethical status of stem cell research and treatment at international and national level. Saudi healthcare vision focuses upon expanding stem cell donation programs and establishing stem cell therapy programs that are medically safe, ethically, and socially approved in the national context [22]. These initiatives are necessary to control the burden of non-communicable diseases and improve the quality of life amongst the Saudi population [23].

The field of regenerative medicine is confronting significant challenges in satisfactory implementation of stem-cell based therapies due to low rates of stem cell donation in various countries and less professional conduct in delivery of stem-cell based treatment by international agencies. The importance of current study is implicit in this local and global context because most of the Saudi citizens who sought stem-cell based therapies are currently relying on international donor agencies for retrieval of stem cells due to extremely low rates of stem-cell donation in the country. Moreover, patients approach international stem cell clinics for stem-cell based treatment due to the non-availability of these treatment facilities in Saudi Arabia and Middle East region. The study has several strengths as it provides first-hand information by conducting a first large-scale survey in Ha’il region of Saudi Arabia. The study questionnaire comprises of simple short items thus provides accurate estimates about levels of awareness and stem-cell donation registration status in target population. The scope of study aligns with the goals of any community health research that focuses on health risk prevention and health promotion. Study provides foundational evidence to develop local health education policies and program to promote stem cell donation behaviors in target population and control the health risks due to lack of information and awareness. Findings need to be interpreted keeping in view some of the study limitations. The research employed a cross-sectional online survey method and collected data from a sub-population in Ha’il city. The study sample comprises of respondents who have completed at least 14 years of formal education in various disciplines and were users of internet thus findings are not applicable to general population of Saudi Arabia. Moreover, participants completed online self-report questionnaire which may contribute to biased response pattern particularly noticed on a question that asked participant’s willingness to donate stem cells. Additionally, the survey assessed the obstacles by asking closed-ended questions with fixed response options and thus did not provide insight about any other hinderances in registration for stem donation. We discussed the findings keeping in view strengths and limitations of study and made some recommendation to increase awareness about stem cell sources, to promote stem cell donation behaviors and directions for future research.

The scientific literature reports that bone marrow is a rich source of CD34 + stem cells that could be used for myocardial repair to prevent heart failure in patients [24]. In our study, a large section of respondents did not consider bone marrow as source of stem cells. This finding has important implication in context of stem cell donation behaviors in Saudi Arabia because the King Faisal Specialist Hospital and Research Centre in Saudi Arabia primarily register donors for bone marrow stem cells transplant. Findings suggest that peoples’ perceptions about fatty tissues as source of stem cells was a significant predictor for willingness to donate stem. These findings have significant implications in context of regenerative medicine in Saudi Arabia, because adipose tissue have the potential to repair, maintain, or enhance various tissues. Moreover, human adipose tissue is ubiquitous and easily obtained in large quantities using a minimally invasive procedure thus retrieval of stem cells from fatty tissue is relatively simpler. Lastly, stem cell retrieved from fatty tissues have several clinical applications such as used to treat fibrosis, atrophy, retraction, and ulcers in patients who underwent radiation therapy [25]. This study validates that awareness about Saudi Stem Cell Registry was significantly associated with willingness to donate stem cells. Findings have significant implications to enhance the awareness through various means of mass communication and aligns with prior literature [26]. In current times, most people seek health information from internet and social media, that is also verified by results from the current survey. It is, therefore, recommended to develop educational and informational programs in consultation with approved agencies under the supervision of the health ministry. Findings showed that means of information significantly associated with willingness to donate and registration status. These findings imply that carefully designed health education programs that share information about authorized sources of stem cells, increase awareness about Saudi Stem Cell donor Registry will be useful to enhance levels of awareness in the community, will protect both donors and recipients of stem cells and will increase the rates of donation.

The survey shows that a higher proportion of respondents demonstrated willingness to donate stem whereas the rates of stem cell registration were low in this study sample. A plausible explanation for this discrepancy is the lack of awareness about donor agencies and procedures which is also flashed as a major obstacle in stem cell registration. This pattern of findings can also be explained in the context of resource dependence theory that explains donation behavior as pro-social behavior and determined by access to several resources including personal, social, and cultural [27]. At a personal level, enhancing awareness and knowledge may contribute positively to promoting stem cell registration. The chi-square analysis showed that having a first-degree relative with experience of stem-cell treatment was significantly associated with registration status for donation of stem cells. This shows that social circumstances may also influence donation behaviors. However, these inferences need to be validated in future research by exploring other social and cultural factors.

The current survey indirectly created an opportunity to make people aware about stem cell donation agencies and procedures by referring them to Donor Education Information Page [19] while asking their intent to donate. This awareness might have contributed to higher levels of willingness to donate in this study sample. Regardless of any contextual factors, the willingness to donate stem cells is a positive sign and it needs to be translated to actual donation behavior by resolving the barriers faced by respondents in stem cell registration.

Regenerative medicine is an emerging science and prior research shows that even health professionals are not well-equipped to guide patients about possibilities of donation, procedures, and agencies. A study from Nigeria reported that dentists have poor knowledge about use of stem cells despite holding positive attitudes [28]. Prior literature reports that medical and nursing students and health workers in Saudi Arabia have low levels of knowledge about various aspects of regenerative therapies and stem cell donation [29]. These findings underscore that public health education policies and programs should focus on propagating information about sources of stem cells, authorized donor agencies which collect stem cells and their procedures.

Our study demonstrated that concerns about impact on health was associated with non-registration for stem cells that aligns with findings from a Malaysian study where attitude about the potential side effects of HSC donation was negatively related with intention to donate among blood donors [21]. It is recommended that information about potential side effects be shared with the public and address their concerns to promote registration for stem cell donation. Moreover, findings indicated that one of the significant factors in non-registration was health conditions that did not allow them to donate stem cells. Apparently, it is true that people with certain health conditions that are experienced at severe levels cannot register for donation of stem cells. However, keeping in view the lack of adequate knowledge about these conditions, there is need to increase the awareness about eligibility criteria for donation of bone marrow and peripheral blood stem cells. Previous literature has demonstrated that development of proper guidelines that educate people with right information about donation risks and benefits along with eligibility are helpful to recruit voluntary hematopoietic stem cell donors [30]. This will help in propagation of accurate information and might help in increasing the registration rates for donation.

The individual and collective behaviors of Saudi people are strictly determined by the religious and cultural values. Individuals seek self-integrity through holding these values. Findings revealed that concerns about religious implications also relate to non-registration for stem cell donation. The field of regenerative medicine has encountered several controversies since the last two decades due to varying perceptions of people about sources of stems cells and heated debates on embryonic stem cell research in academic, political, religious, and public spheres in various regions of the world [31, 32]. Such debates are ongoing and influence the perceptions and donation behaviors of the public. The perceptions related to religious implications need to be addressed because “2003 fatwa” provides the religious framework that permit retrieval and use of stem cells obtained from ethically approved sources. Moreover, the Research Ethics Law decreed in 2010 provides clear ethical guidelines to conduct stem cell research in Saudi Arabia. This information should be widely disseminated to clarify the misperceptions of potential donors. Future research should focus on the identification of specific nature of conflicting views through qualitative data to gain deeper understanding of such hindrances.

## Conclusion

The rates of registration for stem cell donation are very low and need to be improved. Factors such as increasing awareness about sources of stem cells, providing information about operations of local stem cell donor organizations, addressing concerns of people about side impacts of donation and health conditions that might not impact eligibility are some of the useful aspects of information to be shared with people. These educational interventions likely to enhance stem cell registration status among people living in Ha’il city of Saudi Arabia. Findings imply that health education policy and programs should also focus on these aspects to improve the prospects of regenerative medicine in this region of Saudi Arabia.

## Data Availability

The data presented in this study are available on request from the corresponding author with a reasonable reason.
